# Fertility outcome of intravaginal insemination in women with unconsummated marriages

**DOI:** 10.4314/ahs.v24i4.18

**Published:** 2024-12

**Authors:** Rubina Izhar, Samia Husain, Zubaida Masood

**Affiliations:** 1 Department of Gynaecology and Obstetrics Abbasi Shaheed hospital & karachi medical and Dental College Karachi, Pakistan; 2 Department of Gynaecology and Obstetrics Aziz Medical Center Karachi, Pakistan; 3 Department of Gynaecology and Obstetrics Abbasi Shaheed Hospital & Karachi Medical and Dental college Karachi, Pakistan

**Keywords:** Fertility outcome, intravaginal insemination, unconsummated marriages

## Abstract

**Background:**

**Aim:**

To assess the fertility outcome of intravaginal insemination in women with unconsummated marriages.

**Methods:**

We conducted a prospective observational study from January 2017 to October 2020. The study population comprised of couples with unconsummated marriages. Participants were divided into three groups: (1) women with vaginismus (2) men with erectile dysfunction(ED); and (3) men who suffered from premature ejaculation (PE). The couple was educated about the fertility period and advised to perform insemination at home on every alternate day for at least twelve cycles. Pregnancy was defined as a positive urine pregnancy test. The couple was asked to report a positive pregnancy test or follow up at the end of twelve months in case conception did not occur.

**Results:**

The study comprised of 769 couples. There were 371 couples with vaginismus, 258 with premature ejaculation and 140 with erectile dysfunction. Pregnancy was achieved in 270(72.8%) couples with vaginismus, 181(70.2%) with premature ejaculation and 72(51.4%) with erectile dysfunction. The time to conception was shortest for couples with vaginismus, 7.72±3.02 months and longest for couples with erectile dysfunction 10.81±1.53 months.

**Conclusion:**

Couples with unconsummated marriages conceive with intravaginal insemination. At home insemination is a treatment option for such couples before embarking on artificial reproductive techniques.

## Introduction

Unconsummated marriage is commonly encountered in fertility clinics of conservative societies, where sex education is scarce.^1^ The term unconsummated marriage is used when couples are not able to achieve penetrative vaginal sexual intercourse despite multiple attempts. 2 The prevalence is reported to be in the range of 7-63.9 %. 3-5 Common causes in females are vaginismus and dyspareunia, while erectile dysfunction (ED) and premature ejaculation (PE) are the leading causes in males. 6 Vaginismus is the leading cause of unconsummation. Vaginismus leads to involuntary spasm of vaginal muscles, which precludes any penile penetration. 7 Erectile dysfunction is characterized by inability of male partner to attain and maintain an erection suitable for penetration of female vagina.^8^ This problem could be moderate to severe in 5-20% of cases.^9^ In PE, the male partner ejaculates always or almost always before vaginal penetration and is unable to delay ejaculation on all or nearly all vaginal penetrations. The documented prevalence of PE is 20%–30%.^10^

These couples are unable to consummate their marriage and have a hard time conceiving. Although artificial reproductive techniques can be used in these couples, they are expensive and invariably invasive. Intravaginal insemination has been shown to improve conception in these cases. 5 It is simple and low cost option available that can be performed at home by the couple.^11^ Periovulatory vaginal insemination coupled with consistent condom use has been proven to help serodiscordant couples with HIV (Human immunodeficiency virus) get pregnant without risking sexual HIV transmission.^12^ However there are no prospective studies in populations where inability to have intercourse is the only problem. We conducted this study to assess the fertility outcome of intravaginal insemination in women with unconsummated marriages.

## Methods

We conducted a prospective observational study from January 2017 to October 2020. The study population comprised of couples with unconsummated marriages. A non-probability consecutive sampling technique was used for recruitment. Couples were eligible if they had Unconsummation of marriage. The couples were included if the male partner's semen analysis was normal according to World Health organization criteria, transvaginal ultrasound of the female partner was normal, i.e. she did not have any fibroids, polyps or any ovarian cysts on the scan. Women who had PCOS, defined according to Rotterdam criteria, women with thyroid disorder, hyperprolactinemia and non-consenting women were excluded.

Couples were counseled about their issue and management options were discussed with them. Couples who met the inclusion criteria were counseled by trained health professionals and told about the benefits of IVI in their situation and emphasis was placed on trying a low cost and less invasive method. Participants were given a choice to perform intravaginal insemination (IVI) themselves or have intrauterine insemination at the clinic. The methods available were introduced in an unbiased manner. Participants were thus enabled to make an informed choice. Couples who opted for IVI were asked to participate in the study. Consenting couples were offered information leaflets.

Couples were divided into three groups: (1) couples where woman had vaginismus; (those couples, in which the woman with vaginismus had a male partner with premature ejaculation or erectile dysfunction, were excluded) (2) couples where male partner had erectile dysfunction; and (3) couples where male partner suffered from PE. They were explained the procedure by a trained healthcare provider (with an experience of 15 years) in a dedicated area in the outpatient department. We instructed the male partner to collect semen by masturbation in any sterile container. The semen was then drawn up in a 3 or 5 ml syringe barrel. He was taught to introduce this syringe into the vagina of the female and deposit the semen into vagina by pushing the plunger gently. We advised the female to lie down on a pillow under her back and the male was instructed to position the syringe at an angle of least 45 degrees for proper deposition. To avoid seepage we asked the female lie supine for 20 minutes at least. The couple was educated about the fertility period and advised to perform insemination at home on every alternate day for at least twelve months. The fertile period was calculated for each couple individually, e.g. for a woman with shortest menstrual cycle of 24 days, subtracting 18 days from the shortest cycle would mark the first day of her fertile period (i.e. day 6), similarly, for the same woman if the longest cycle was 31 days, subtracting 11 days from the longest cycle would mark the last day of her fertile period (i.e. day 20). So, this woman would be most fertile between days 6 to 20 of her menstrual cycle.

The pregnancy was defined as a positive urine pregnancy test. The couple was asked to report a positive pregnancy test. However if the conception did not occur, they were given an appointment to discuss further options.

The quantitative variables, duration of marriage and duration from treatment to conception were presented by means and standard deviation. One way anova was used to compare means for groups. Frequency and percentages were computed for qualitative variables; age range and conception. Chi square test was used to compare the groups.

Survival analysis was performed to evaluate the probability of conceiving in all three groups. Curves were compared by means of mantel Haenszel log-rank test for categorical variable, which in this case was pregnancy. Data were analyzed using SPSS Statistics for Windows, version 15.0 (SPSS, Chicago, IL, USA).

Data were coded to ensure confidentiality. No subjects were harmed, confidentiality was maintained and no subject was enrolled in the study without formal informed consent. The study was carried out in accordance with the Declaration of Helsinki. The hospital head supervised the project. The study was approved by the institutional review board (IRB 006-2016).

## Results

During the study period, 787 couples satisfied the inclusion criteria and were asked to participate. Of these 8 refused to participate and 10 were lost to follow up. The study therefore comprised of 769 couples. There were 371 couples with vaginismus, 258 with premature ejaculation and 140 with erectile dysfunction. Most were less than 35 years old. Pregnancy was achieved in 270(72.8%) couples with vaginismus, 181(70.2%) with premature ejaculation and 72(51.4%) with erectile dysfunction. The time to conception was shortest for couples with vaginismus, 7.72±3.0 months, and longest for couples with erectile dysfunction 10.81±1.53 months. Table 1

**Table 1:** characteristics of groups

	Cause of Unconsummation of marriage N=769			P value*
	Vaginismus n=371	Premature ejaculation n=258	Erectile dysfunction n=140	
	n (%)	n (%)	n (%)	0.963
Age				
less than 35 years	299(80.6%)	203(78.7%)	111(79.3%)	
36-40	47(12.7%)	37(14.3%)	18(12.9%)	
41 and above	25(6.7%)	18(7.0%)	11(7.9%)	
				
Time to conception	7.72±3.02	9.44±2.09	10.81±1.53	0.001**
Duration of marriage	3±2	4±2	4±2	0.362
PREGNANCY				0.001*
yes	270(72.8%)	181(70.2%)	72(51.4%)	
no	101(27.2%)	77(29.8%)	68(48.6%)	
				

* The Chi-square statistic is significant at the 0.05 level.

** t-test Anova between groups is significant at the 0.05 level

Age was statistically associated with conception in two sub groups. Table 2 women aged less than 35 years were significantly more likely to conceive in vaginismus and PE but not erectile dysfunction.

**Table 2:** Startification according to age of conception in groups

	CAUSE								
	Vaginismus			Premature ejaculation			Erectile dysfunction		
	PREGNANCY			PREGNANCY			PREGNANCY		
	yes	no	P value	yes	no	P value	yes	no	P value
age	N %	N %		N %	N %		N %	N %	
less than 35 years	232 (77.6%)	67 (22.4%)	0.001*	159 (78.3%)	44 (21.7%)	0.001*	61 (55.0%)	50 (45.0%)	0.173
36-40	29 (61.7%)	18 (38.3%)		16 (43.2%)	21 (56.8%)		8 (44.4%)	10 (55.6%)	
41 and above	9 (36.0%)	16 (64.0%)		6 (33.3%)	12 (66.7%)		3 (27.3%)	8 (72.7%)	

* The Chi-square statistic is significant at the 0.05 level

**Figure 1:**
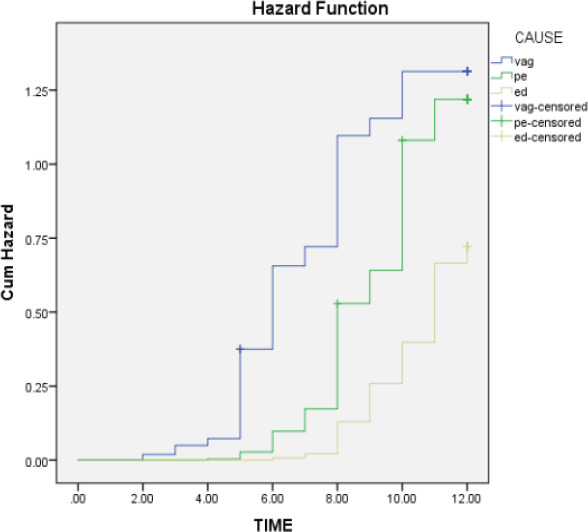
Cumulative pregnancy rate in different group *Log Rank (Mantel-Cox) ≤0.001

Time to conception in different groups was also statistically significant. Figure 1 Women with vaginismus got pregnant earlier than couples where the primary problem was erectile dysfunction or premature ejaculation.

## Discussion

Our study shows that couples with unconsummated marriage due to sexual dysfunction conceive with intravaginal insemination. Moreover, the results indicate that women with vaginismus are likely to get pregnant sooner than couples where the male partner has erectile dysfunction or premature ejaculation.

Intravaginal insemination has been used as a method to achieve conception in cases of spinal cord injury in males^13^ and general sexual dysfunction 14. It has also been studied in HIV care among discordant couples as a safer conception method to reduce horizontal transmission.^12^ A retrospective study from India provided pregnancy rates for artificial intravaginal insemination in cases of unconsummated marriages.^11^ Our study provides prospectively collected data on the same subject. Furthermore we calculated time to conception in such cases.

Nonconsummation of marriage in Muslim world is also very common 15. Women may have phobia of vaginal penetration that leads to unconsummation of marriage.^16^ These couples are not very keen on seeking treatment due to the stigma associated with the diagnosis.^17^ By the time they seek treatment, these treatments may lead to suboptimal results because of natural decline in fertility. In our study women who were less than 35 years of age were more likely to conceive in vaginismus and PE but not erectile dysfunction.

Couples where female partner had vaginismus had the best conception rate; 72.8%, followed by couples where male partner had premature ejaculation (70.2%). This pregnancy rate in couples with vaginismus after 1 year was similar to the study from India that had a retrospective design^11^. However, that study reported pregnancy rates after six cycles of insemination. On the contrary, the conception rate for premature ejaculation was only 10.9% in that study, which was lower than couples where male partner had erectile dysfunction 21.8%. In our study, the rates were higher. This could also be attributed to the small size of the study. Moreover, most of the couples had vaginismus (37/55) and only 6 couples with premature ejaculation were included. This may be a reason of lower conception rates.

In cases where the primary cause was erectile dysfunction pregnancy was achieved in 52 % of the cases. This is in agreement with previous reports where 40.3 % males with sexual dysfunction^12^ and 42.3% men with spinal cord injury got their partner pregnant.^11^ The reason for lower conception could be inadequate or impossible collection of semen as all inseminations were conducted at home. However, these numbers were comparatively lower than those reported by Leduc (90%) where intravaginal insemination was done at clinics and vibrators and other devices were used to collect semen.^18^

The time to conception was different in all groups. Women with vaginismus got pregnant earlier than couples where the primary problem was erectile dysfunction or premature ejaculation. This could be explained partially by inadequate collection of sample to inseminate. We therefore suggest that couples with premature ejaculation and erectile dysfunction should be offered insemination at clinics in cases where conception is not achieved within first six months.

Artificial home vaginal insemination, though inexpensive, may not yield the same results in such cases and may need longer commitment and compliance with the treatment methods. Because this procedure can be easily done at home, it holds a special place in situations where privacy is of prime importance to the couple.

It may be argued that intrauterine insemination should be offered to patients with unconsummated marriage. We would counter argue that for couples where the cause is vaginismus, a trial of intravaginal insemination should be offered. Others should be counseled that it may take longer and would be more successful if performed in clinic.

## Conclusion

Couples with unconsummated marriages conceive with intravaginal insemination. At home insemination is a treatment option for such couples before embarking on artificial reproductive techniques.
